# Lingering Effects of Early Institutional Rearing and Cytomegalovirus Infection on the Natural Killer Cell Repertoire of Adopted Adolescents

**DOI:** 10.3390/biom14040456

**Published:** 2024-04-09

**Authors:** Elizabeth K. Wood, Brie M. Reid, Dagna S. Sheerar, Bonny Donzella, Megan R. Gunnar, Christopher L. Coe

**Affiliations:** 1 Department of Psychiatry, Oregon Health & Science University, Portland, OR 97239, USA; 2 Department of Psychiatry and Human Behavior, Brown University, Providence, RI 02906, USA; brie_reid@brown.edu; 3 Wisconsin Institute of Medical Research, University of Wisconsin Comprehensive Carbone Cancer Center, Madison, WI 53706, USA; dsheerar@wisc.edu; 4 Institute of Child Development, University of Minnesota, Minneapolis, MN 55455, USA; donzella@umn.edu (B.D.); gunnar@umn.edu (M.R.G.); 5 Department of Psychology, University of Wisconsin-Madison, Madison, WI 54706, USA; ccoe@wisc.edu

**Keywords:** adolescence, adoption, adversity, cytomegalovirus, infancy, inflammation, immunophenotyping, natural killer cell, orphanage, tumor necrosis factor

## Abstract

Adversity during infancy can affect neurobehavioral development and perturb the maturation of physiological systems. Dysregulated immune and inflammatory responses contribute to many of the later effects on health. Whether normalization can occur following a transition to more nurturing, benevolent conditions is unclear. To assess the potential for recovery, blood samples were obtained from 45 adolescents adopted by supportive families after impoverished infancies in institutional settings (post-institutionalized, PI). Their immune profiles were compared to 39 age-matched controls raised by their biological parents (non-adopted, NA). Leukocytes were immunophenotyped, and this analysis focuses on natural killer (NK) cell populations in circulation. Cytomegalovirus (CMV) seropositivity was evaluated to determine if early infection contributed to the impact of an atypical rearing. Associations with tumor necrosis factor-alpha (TNF-α) and interferon-gamma (IFN-γ), two cytokines released by activated NK cells, were examined. Compared to the NA controls, PI adolescents had a lower percent of CD56^bright^ NK cells in circulation, higher TNF-α levels, and were more likely to be infected with CMV. PI adolescents who were latent carriers of CMV expressed NKG2C and CD57 surface markers on more NK cells, including CD56^dim^ lineages. The NK cell repertoire revealed lingering immune effects of early rearing while still maintaining an overall integrity and resilience.

## 1. Introduction

Human infants, as well as the offspring of many animals, are born with the expectation of a supportive rearing environment and evince a strong motivational need to identify their primary care provider(s) [1]. If these biological and emotional expectancies are not met due to separation and loss, or if the parental care is not responsive and nurturing, it can have deleterious effects on neurobehavioral development even when adequate nutrition and hygiene are provided [2,3,4]. In addition to increasing emotional reactivity, impairing attentional processes, and undermining cognitive potential, there can be cascading effects on many physiological systems. Short-term and lasting alterations in neuroendocrine and metabolic functioning have been identified in both animals and humans, highlighting the many ways that early life adversity (ELA) can increase the risk for poor health later in life [5,6,7,8,9]. While the vulnerabilities engendered by ELA have been extensively documented, less research has focused on the capacity for recovery if rearing conditions improve during development.

At the psychosocial level, receipt of parental warmth later in childhood and the experience of positive social relations during adolescence and adulthood can mitigate some long-term effects, enabling resilience despite earlier adversity [10,11]. In addition, regulatory and constitutive processes in many physiological systems may limit the progression from early adversity to overt pathophysiology and disease, even when subclinical changes in set points, signaling, and function remain detectable [12,13,14,15,16]. These more subtle changes are often interpreted as a type of allostatic shift and are indicative of a new adaptive homeostasis [17,18,19]. The following research investigated whether there is evidence for this type of persistent recalibration within the immune system after the resolution of adverse rearing conditions during infancy. Leukocyte profiles were evaluated in adolescents who had spent their infancies in institutional settings but were then adopted and raised by supportive and well-resourced families.

The impact of ELA on inflammatory physiology and the immune system has been the focus of many investigations over the last four decades [20,21,22]. This research documented that there are often chronic increases in the circulating levels of proinflammatory cytokines such as interleukin-6 (IL-6) and subclinical elevations in C-reactive protein (CRP), an acute phase reactant secreted by the liver [22,23,24]. Prolonged changes in inflammatory activity after ELA have been observed both in individuals who experienced neglect and deprivation during infancy and in adults who had suffered from childhood maltreatment at an older age [25,26]. Coincident with the increased release of soluble inflammatory mediators, inhibitory effects on humoral and cellular immune responses have been reported [27,28]. Proliferative responses are usually reduced when lymphocytes are cultured in vitro and stimulated with mitogens or endotoxin. Similarly, the actions of cytolytic lymphocytes are usually smaller and less effective against virally transformed and mutagenic target cells. In addition, ELA can impair defenses against bacterial and viral infections and undermine the immune control needed to prevent the reactivation of herpes viruses that remain latent in tissue following the primary infection [29].

Technological advances in immunophenotyping methods and a more complete understanding of the proteins, receptors, and other membrane molecules on the surface of leukocytes now permit deeper insights into the cellular processes accounting for immune alterations after ELA. For example, in the adopted adolescents who are the focus of the current analysis of natural killer (NK) cells, we found that institutional rearing during infancy led to there being more terminally differentiated T cells in circulation [30]. A similar effect on specific T cell populations was found in young adults who had been adopted during early infancy [31]. The accumulation of replicatively senescent T cells is seen normally in elderly adults, which suggests that ELA could result in accelerated cellular aging [32]. This interpretation would appear to be supported by the epigenetic modifications and changes in gene transcription detected in mononuclear cells (MNC) [33,34,35]. In addition, the NK cells of young adults who spent the first several months of their infancy in orphanages before being adopted were found to have lower levels of the intracellular factors involved in degranulation and cytolytic activity, including perforin and granzyme B [36]. The following analysis extends the investigation of how ELA affects NK cell biology with a more in-depth examination of the many lineages within the NK cell population.

In addition, we investigated whether exposure to CMV is a contributing factor to the immune alterations because the communal conditions of orphanages result in a high prevalence of CMV infection among both the children and adult care providers [37,38]. CMV is never entirely cleared from the body and persists as a lifelong latent infection of hematopoietic progenitor cells and monocytes. Infection is also a concern because it can precipitate a type of immune imprinting, biasing immune responses and taking a toll on the immune system because of the cellular effort needed to keep the virus from reactivating [39,40]. Recent research has shown that CMV seropositivity is associated with lower NK cell responses and can reduce the immune protection generated by some but not all vaccines [41].

Historically, the importance of NK cells was first recognized because of their role in identifying and lysing virally infected cells and mutagenic tissue [42]. However, it is now appreciated that they also perform other essential functions during development. NK cells derive from stem cell progenitors early during the first trimester and are functional at the time of birth. They are integral to the innate immune defenses that protect the neonate prior to the maturation of adaptive immune responses that rely on antigen presenting cells and the postnatal priming of T cells by antigens that is required for their functional engagement [43,44]. At the microscopic level, all NK cells are visually similar and appear to be large, granular lymphocytes. However, with modern immunophenotyping, many different NK cell lineages can be delineated with a diverse array of proteins, receptors, and signaling molecules on their surface [45,46,47].

As a group, NK cells are recognized by the absence of CD3, the surface protein complex and co-receptor that defines T cells. These CD3− lymphocytes also typically express an identifying adhesion molecule on their surface, CD56, which facilitates cell-to-cell interactions (i.e., the neural cell adhesion molecule, NCAM-1). Based on the expression of CD56 at a high or low density, NK cells are differentiated into two primary subsets [48]. This dichotomy of CD56^bright^ and CD56^dim^ NK cells, respectively, has functional significance. CD56^bright^ NK are thought to be less mature, are more commonly located in tissue, including the spleen and liver, and are prone to synthesize cytokines, enabling them to modulate adaptive immune responses [49]. In contrast, the CD56^dim^ NK population predominates in the blood stream, where they account for about 90% of circulating NK cells, and they are more predisposed to engage in cytolytic responses [50,51,52]. Our immunophenotyping panel was designed to identify the percentages of these two major subsets of NK cells and to determine whether a history of ELA differentially affected the CD56^bright^ or CD56^dim^ NK population.

NK cells can be differentiated further by the presence or absence of other surface proteins, including CD16, a low affinity, activating receptor for immunoglobulin (FcγRIII) that facilitates the recognition of antibody-coated, opsonized targets [53]. This membrane receptor is present on most CD56^dim^ NK cells and facilitates antibody-dependent cell cytotoxicity (ADCC). In addition, NK cell lineages are identified by the presence of other proteins and epitopes that reflect the cell’s activation history and are indicative of the propensity to proliferate. We optimized the immunophenotyping panel to enumerate NK cells co-expressing two additional surface proteins: NKG2C, an activation receptor involved in pathogen recognition, and CD57, a surface epitope indicative of mature NK cells. While NK cells with these surface markers are still predisposed to engage in cytolytic activity, these cells are thought to have reached a stage of terminal differentiation with a reduced capacity to replicate [54,55].

The NK cell repertoires of adolescents who had history of ELA were compared to the profiles of age-matched healthy controls raised in birth families with educational backgrounds and incomes that were like the families who adopted. The primary aim was to test whether the early adversity of institutional rearing had a selective and more specific influence on the CD56^bright^ and CD56^dim^ NK cells, given that these two lineages have different distribution patterns between residence in tissue, adherence to the walls of blood vessels and airways, and trafficking in the blood stream. The prevalence of CMV and herpes simplex virus (HSV) infection was assessed to determine if being a latent carrier of herpes viruses contributed to the immune effects of ELA. Based on the clinical literature, our a priori hypothesis was that CMV seropositivity would be associated with an expansion of NK cells expressing NKG2C and CD57 [56,57]. In addition, the possible associations of the NK cell profile with tumor necrosis factor-alpha (TNF-α) and interferon-gamma (IFN-γ) levels were evaluated because both are prominent Th1-type cytokines synthesized by activated NK cells [58]. They are also sentinel indicators of proinflammatory activity. Many studies have found that cytokine levels are altered after ELA as well as in depressed and anxious adolescents who had a childhood history of trauma [20]. The adopted adolescents in our study were evaluated over a decade after the adversity of their early institutional rearing had ended.

To elucidate the lingering effect of ELA on the NK cell repertoire, we first assessed differences in the percent of NK cells expressing CD56 at a high or low density among the post-institutionalized (PI) adolescents and healthy non-adopted controls (NA). Then, CD3−CD56+ NK cells expressing the CD16 surface marker and co-expressing NKG2C and CD57 were examined. Given the lasting impact of early CMV infection on immunity and the high rates of CMV seropositivity when children are raised in group settings, another goal was to clarify the mediating role of CMV infection on the association between early rearing and NK cells. Finally, we considered whether the persistent effects of early rearing on NK cells were associated with later stressful life events and negative affect or mediated by differences in basal and stimulated TNF-α and IFN-γ secretion.

## 2. Materials and Methods

### 2.1. Participants

Blood samples were obtained from 45 adolescents initially raised in institutional settings outside the United States but then adopted by American families between 5 and 45 months of age (median 12.5 months; post-institutionalized, PI). Their immune profiles were compared to 39 age-matched, healthy American controls raised by their biological families (non-adopted, NA). Blood sampling occurred from 2015 to 2017. At the time of blood collection, which was prior to the COVID pandemic, the participants were between 13 and 21 years of age (mean [SD] = 16.3 +/− 2.01 years). PI youth were recruited from a registry of families who had adopted internationally. The NA youth were also recruited from a registry, comprised of birth families willing to be contacted about participating in research. Additional information about this cohort has been provided in prior publications, which reported on their neuroendocrine physiology, cytokine levels, and T cell biology [15,30,59]. Exclusion criteria included: major congenital abnormality, a prior diagnosis or signs of fetal alcohol exposure (FAE), a diagnosed immune disorder, current use of steroidal prescription medication, and a combination of an elevated leukocyte count above 15.0 × 10^3^/µL and C-reactive protein (CRP) over 10 mg/L. Four of 49 recruited PI participants were excluded from the data analysis. One participant was excluded due to possible FAE, two participants had autoimmune conditions, and one participant had high WBC and CRP indicative of a recent infection. The exclusions resulted in a final *n* of 45 PI adolescents.

This study was reviewed and approved by Institutional Review Boards at the Universities of Minnesota and Wisconsin (IRB # 1503M66241, approved 4 March 2015), and the protocol adhered to the Declaration of Helsinki guidelines. Parents provided informed consent for their children to participate. Participants over 18 years also consented, while those under 18 years of age assented to all procedures and were aware they could decline and withdraw at any time.

### 2.2. Familial Resources

Maternal educational attainment was reported on a categorical scale through college degree and post-graduate training. In addition, to verify that the family households of PI and NA youth had similar resources, familial income was reported on a tiered scale ranging from a low of $25,000 to >$200,000.

### 2.3. General Health

Health questionnaires were completed by parents and participants at home. These reports focused on early childhood health when PI youth were either still in the orphanage-like institutions or recently adopted, and then subsequently on childhood and adolescence when both PI and NA participants lived in similar family situations. In addition, parents completed a Health Index report on any recent illness in their children during the prior month. Finally, participants completed the Adolescent Health Habits Questionnaire, a 79-item instrument designed for youth between 12 and 18 years of age. It focuses on general health, asthma, allergies, recent infections, sleep habits, daytime sleepiness, diet, alcohol, and medication use.

To assess for exposure to traumatic experiences following institutionalization, a modified version of the life events checklist (LEC) was also administered to parents of participants [60,61]. For PI participants, parents completed the LEC for events that happened since the child was adopted. For NA participants, parents completed the checklist for events that happened in the past year. The LEC is a 37-item measure that captures the number and impact of stressful or difficult life events that a child or adolescent has experienced in the past year. Parents reported participant experiences with stressful life events (e.g., addition of a sibling to the family, death of close family member, divorce or separation of parents) and how much it impacted their child’s life, rated on a scale of 0 (none) to 4 (a great deal). The LEC score reflects the sum of all events and their impact or the sum of all risk-related events and their impact. Here, we examined the sum of LEC risk-related events.

To assess psychological status at the time of the blood draw, participants completed the mental health symptom section of the MacArthur Health and Behavior Questionnaire (HBQ-C v2.1 for Late Childhood and Adolescents) [62,63,64]. The HBQ-C has 38 items and captures a range of mental health symptoms; asking children to rate themselves by the degree to which statements apply to them (for example, “I’m a sad person”). The HBQ-C has strong psychometric properties and is designed to assess mental health in childhood through adolescence [65,66]. The HBQ-C was completed by the participants without the parent present. Participant HBQ-C scores for total internalizing symptoms, calculated from the subscales for depression (15 items), generalized anxiety (15 items), and separation anxiety (8 items) were used in analyses.

### 2.4. Blood Collection

Blood (25 mL) was collected by a licensed phlebotomist at a university research center, with 10 mL drawn into sodium heparin-coated vacutainers and 15 mL into EDTA-coated vacutainers. All blood was obtained between 0800 and 1300. Mean time of blood collection did not differ between the PI and NA participants (1059 [+/−116 min] and 1053 [+/−110 min], respectively). Fresh samples were shipped overnight with a coolant block for processing and immunophenotyping within 24 h of collection. Analysis of cell viability on the flow cytometer and the complete blood count verified there had not been significant cell loss. Height and weight measures were acquired at the time of blood collection in addition to verifying that body temperature and blood pressure were in the normal range. The staff who oversaw the biomarker testing were not informed of the participant’s early life history.

### 2.5. Immune Measures

#### 2.5.1. Complete Blood Count

One mL of EDTA-treated blood was delivered to a CLIA-certified clinical laboratory (Unity Health-Meriter Labs, Madison, WI, USA) to determine a complete white blood cell count (WBC) and cell differential, including percent and number of lymphocytes and neutrophils.

#### 2.5.2. Immunophenotyping

**Figure A1:**
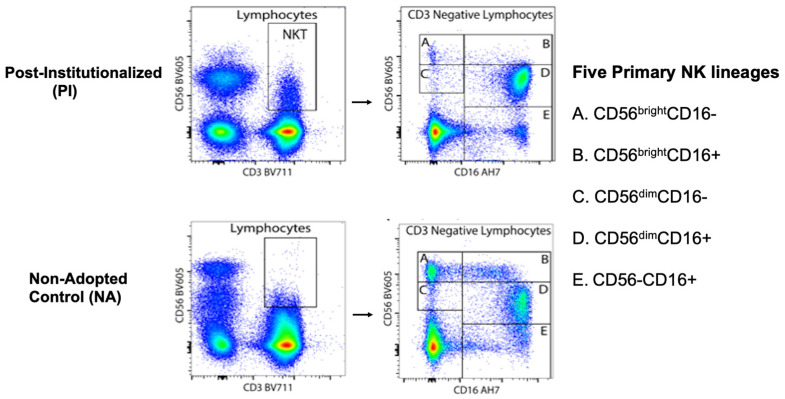
Gating strategy employed to identify the five primary NK subsets used in the analyses. This illustration is based on the immunophenotyping of blood from one participant in each rearing condition. Note the difference in the number of cells and color intensity in the A and B quadrants, which portray the CD56^bright^CD16− and CD56^bright^CD16+ populations, respectively. The PI participants had a significantly lower percent of the CD56^bright^ NK cells in peripheral circulation than the NA participants. Data collection was set to stop after reaching a criterion of 50,000 live, single cells for each sample.

Heparinized blood (7 mL) was analyzed at the Flow Lab in the UW Comprehensive Carbone Cancer Center, where an optimized 18-color panel of monoclonal antibodies (MoAb), fluorochromes, and other standardized reagents was used to characterize leukocyte profiles, including NK cells among the CD3− lymphocytes. Some fluorochrome-antibody combinations were modified from the published protocol to accommodate the BD LSR Fortessa configuration (BD Biosciences, San Jose, CA, USA) [67]. Data collection was set to stop after reaching a criterion of 50,000 live, single cells for each sample. Cells were gated first for viability, followed by gating to distinguish CD3− from CD3+ MNC. The live gate included all cells negative for Ghost Dye (TM) Violet 510; the singlet gate excluded all aggregates based on forward scatter area plotted against forward scatter height, and a cell gate was drawn to identify MNC. Five primary NK cell populations were discriminated by the presence and density of CD56 and CD16 expression among the CD3− lymphocytes (i.e.,CD56^bright^CD16−, CD56^bright^CD16+, CD56^dim^ CD16−, CD56^dim^CD16+, CD56−CD16+), with additional lineage delineation by the presence or absence of the NKG2C and CD57 surface markers. The gating strategy is illustrated in Figure A1 in Appendix A.

#### 2.5.3. Cytomegalovirus (CMV) and Herpes Simplex Virus (HSV) Antibody

CMV seropositivity was determined by quantifying antibody levels using an enzyme-linked immunosorbent assay (ELISA, DRG International, Springfield, NJ, USA). A reference curve was generated from 3 calibrators with known CMV-specific IgG concentrations. In addition to determining CMV antibody level, the adolescents were categorized as being either latently infected with CMV or virus-negative. Following the manufacturer’s instructions, values higher than the second calibrator were designated as CMV-seropositive.

HSV antibody titer was measured in duplicate determinations by ELISA (HerpeSelect IgG, Focus Diagnostics, Cypress, CA, USA). IgG level was quantified with respect to a reference curve generated from 5 calibrators. The assay protocol included low and high positive controls (1.5–3.5 and >3.5, respectively), as well as a negative control (0).

#### 2.5.4. Cytokine Levels and Cell Culture Stimulation

Cytokines were assayed in multi-analyte arrays using an electrochemiluminescence platform (MesoScale Discovery, Rockville, MD, USA). The current analysis focuses on 2 cytokines, TNF-α and IFN-γ, from a 10-cytokine panel. These two cytokines were selected because they are prominently released by activated NK cells, and both polarize for Th1-type immune responses. The lower limit of detection (LLOD) was below 1 pg/mL, but importantly, this assay has a broad dynamic range with upper detection to the ng/mL range. Intra-assay coefficients of variation for the duplicate determinations were below 10%.

In addition to determining basal cytokine levels in plasma, leukocytes were stimulated in whole blood cultures in vitro to assess cellular reactivity. For this analysis, a cocktail of phorbol myristate acetate and ionomycin (PMA/Io) was selected as the stimulant condition because it is a robust stimulator of NK cells and quickly elicits the release of TNF-α and IFN-γ, the two cytokines of interest. The heparinized blood was mixed with an equal volume of media (IDM, MEM, and an antibiotic-antimycotic solution [Gibco]). Then, 1 mL of this 1:1 solution was added in triplicate replicates to a sterile 24-well culture plate and incubated with PMA/Io (10 ng/1 u/mL) for 4–5 h at 37 °C with 5% CO_2_. One unstimulated well was used as a control to monitor spontaneous release of cytokines. Supernatants were harvested, spun at 2500 rpm for 8 min, frozen in cryovials, and stored in an ultracold freezer at −70 °C until thawed for the cytokine assay. Supernatants from the stimulated cell cultures were diluted with the manufacturer’s assay media so that cytokine values were on the linear portion of the reference curve and below the upper limit of quantification (ULOQ). Some findings related to TNF-α in adopted adolescents were reported previously, but only as a composite score with IL-1 and IL-6, and the prior analysis focused only on the association with T cells, not NK cells [59]. That analysis also did not include IFN-γ, a prominent cytokine that can both stimulate and be released by NK cells.

#### 2.5.5. C-Reactive Protein (CRP)

CRP levels were quantified with the same electrochemiluminescence platform using a single-plex assay (MesoScale Discovery, Rockville, MD, USA). Plasma was diluted so that the high concentrations of CRP in blood were aligned on the linear portion of the standard reference curve. Quantification was in pg/mL, but CRP values are reported in mg/L units to be consistent with the clinical literature. All samples were analyzed in duplicate determinations. The intra-assay CV was below 10%.

### 2.6. Data Analytic Plan

Descriptive statistics, including means and variance, were examined for the primary predictors and outcome variables to check for normality and the possible need for transformation. Some analyses were conducted twice, both on the original raw data and on adjusted values after log 10 transformation and setting a small number of extreme values to the 3 SD point. The latter adjustment was performed for only 18 out of 2436 values (<1%). Following the recommendations of statistician C.P. Winsor, winsorization (i.e., clipping) was selected over trimming (i.e., omitting) and excluding high values to ensure that unique immune information on all participants was considered [68]. This approach maintains all rank positions but ensures that extreme values do not account for statistical significance.

Chi-squared and *t*-tests, as well as bivariate correlations, were run using SPSS, version 29 (IBM, Armonk, NY, USA, 2022). Path and mediation models were estimated in M*plus*, version 8.6 (Muthen & Muthen, Los Angeles, CA, USA, 1998–2021), using the robust maximum likelihood estimator, which employs a sandwich estimator and accommodates non-normal data distributions. The analytic plan and steps were as follows:

1. Sample description: For the demographic and general health variables, participants from the two rearing conditions were compared with independent *t*-tests for continuous variables. The chi-squared test was used to evaluate differences in categorical variables, including that the representation of females and males was equivalent in both conditions.
2. NK cell subsets in PI and NA adolescents: Path models were used to assess the percent of NK cells expressing CD56 at high and low density (i.e., CD56^bright^ and CD56^dim^) in the blood of PI and NA adolescents. Then, differences in the percent of NK cells expressing both CD56 and CD16 surface markers were examined (i.e., CD56+CD16− or CD56+CD16+). Finally, the co-expression of NKG2C and CD57 was evaluated for each of the 5 primary NK cell subsets. All analyses took sex of the participant, time of blood collection across the morning, and participant age into consideration. The Benjamini-Hochberg (BH) procedure was used to correct for multiple comparisons. Cohen’s *F*^2^ was also calculated to interpret the effect sizes of significant differences between the two rearing conditions, where *F*^2^ values of ≥0.02, ≥0.15, and ≥0.35 reflect small, medium, and large effect sizes, respectively.
3. CMV mediation of effect of early rearing on NK cell: To test whether CMV seropositivity was statistically associated with differences in NK cell percentages and expression of surface membrane molecules, path models were generated in which each NK cell lineage was simultaneously regressed on the participant’s early rearing condition and CMV antibody level. In the same model, CMV antibody titer was regressed on the two rearing conditions. Mediation was tested using the model indirect command in Mplus. Calculated estimates and accompanying confidence intervals were based on 10,000 bootstrapped replications. All models considered sex of the participant, time of blood collection during the morning, and participant age at time of assessment.
4. Associations between early rearing, NK cells, and cytokine levels: The effects of early rearing on basal levels of TNF-α and IFN-γ levels as well as after in vitro stimulation with PMA/Io were then examined. To interrogate the strength of specific associations with NK measures, bivariate correlations between the two cytokines, the 5 primary NK cell subsets, and CMV antibody titer were also examined using Pearson’s test.

## 3. Results

### 3.1. Demographics

Sociodemographic factors, including maternal educational attainment and familial income, did not differ between the two rearing conditions. Most of the mothers had obtained college or postgraduate degrees. The $100,000-to-125,000 category was commonly reported although the median financial level was in the $70,000-to-85,000 range. All parents identified as White. NA participants identified as White (89.7%), Black or African American (5.1%), or more than one race (5.1%), while PI participants identified as White (57.8%), Asian (26.7%), Indigenous of the Americas (4.4%), more than one race (4.4%), or unknown (6.7%). The PI participants had been adopted internationally, mostly from eastern Europe. Thirty (66.7%) were from eastern Europe and Russia; 6 (13.3%) from South Asia; 2 (4.4%) from Latin America; and 7 (15.6%) from Southeast Asia. Median age of adoption was 12.5 months with a range from 5 to 45 months. Participants were between 13 and 21 years of age at the time of blood collection. As shown in Table 1, the mean ages of 16.0 and 16.6 years for the PI and NA participants, respectively, did not differ. Females and males were similarly represented in both rearing conditions. To consider the influence of other traumatic events that could have occurred later during childhood as well as the more recent effect of negative emotions and stress on NK cells, additional information was acquired with the LEC and HBQ-C. Because neither score differed between the PI and NA participants, statistical modeling of the factors affecting NK cell profiles focused on differences in early rearing, CMV infection, and cytokine levels.

### 3.2. General Health

More illnesses were reported for PI participants during early childhood (*t* (82)= 2.73, *p* = 0.008), but their recent health status was like the NA controls. Following adoption, PI participants had a normal exposure rate to distressing life events, and their LEC scores did not differ significantly from the NA controls. In addition, the internalizing domain of the HBQ-C, which was derived from the depression and anxiety subscales, did not differ significantly between PI and NA participants. In keeping with the recruitment strategy, which excluded potential participants with infectious or chronic illnesses, the total WBC, lymphocyte and neutrophil percentiles, and CRP levels were in the normal range (Table 1). While the current health status of the adolescents in both rearing conditions was similar, the PI participants were on average 7 cm shorter at assessment (*t* (82) = −3.30, *p* = 0.001). PI youth were also significantly more likely to be seropositive for CMV (91.1% vs. 35.9%, *p* < 0.001) and had significantly higher CMV-specific IgG levels (*t* (82) = 4.39, *p* < 0.001). Based on the HSV-specific IgG levels, the prevalence of HSV infection among PI and NA adolescents was not as disparate (26.6% vs. 12.8% were HSV-seropositive, respectively). In addition, the HSV-specific IgG level in PI participants was only marginally higher (*p* < 0.06). Thus, subsequent analyses examining whether infection with herpes viruses affected the NK cell repertoire focused only on CMV. However, in Appendix A, we show that there was an independent association between CMV antibody titer and the experience of stressful life events based on the LEC and an association between higher HSV antibody level and the internalizing negative domain of the HBQ-C (Figure A4 and Figure A5, respectively).

### 3.3. Early Rearing and NK Subsets

**Figure 1:**
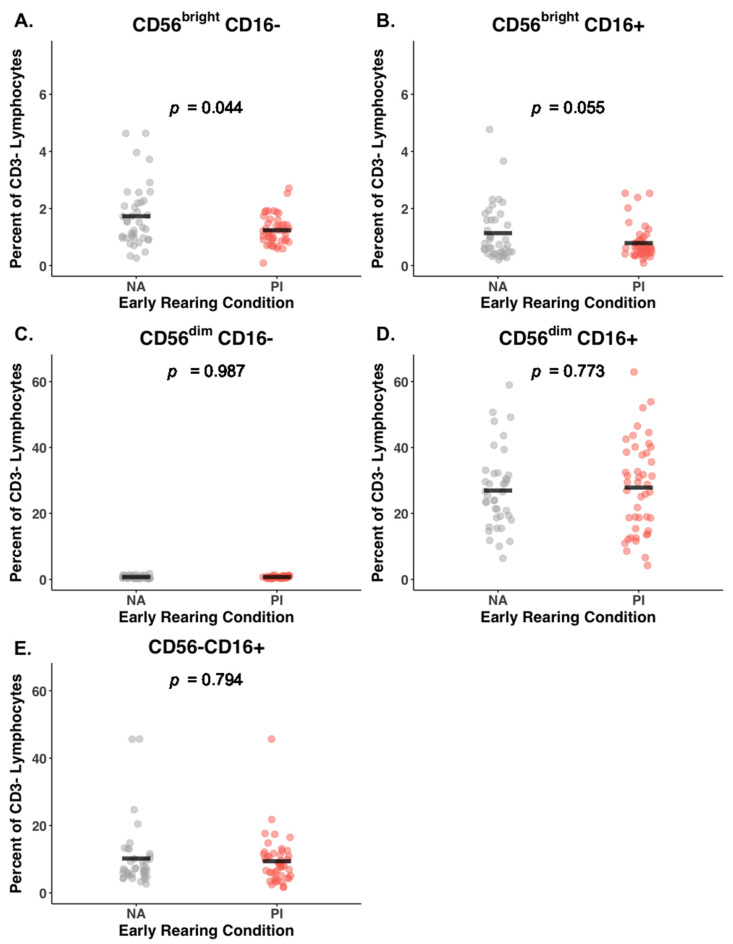
Blood levels of the five primary NK cell subsets identified by immunophenotyping in non-adopted (NA) and post-institutionalized (PI) adolescents. Mean values for each subset are shown in Panels (**A**–**E**) as horizontal black bars. Percentages were determined with respect to lymphocytes gated as being CD3−. Although not the predominant NK subset in the blood, CD56^bright^ NK cell lineages were lower in the blood of PI adolescents (Panels **A** and **B**). The CD56^dim^CD16+ cells accounted for >90% of NK cells in blood, and the percentiles did not differ by early rearing condition until additional surface markers were considered (Panels **C** and **D**). The CD56−CD16+ NK cells did not differ between the two rearing conditions (Panel **E**).

PI adolescents had a lower percent of CD56^bright^ cells in circulation compared to NA adolescents (2.03% vs. 2.93%; BH corrected *p* = 0.02, Cohen’s *F*^2^ = 0.08). Raw and corrected *p*-values are provided in Table 2. As illustrated in Figure 1A,B, this effect of early rearing on the CD56^bright^ NK cells was evident for both the CD56^bright^CD16− and CD56^bright^CD16+ NK cells, although only the lower percentiles for the CD56^bright^CD16− NK lineage remained above the significance threshold after correction (PI: 1.23%, NA: 1.73%; BH corrected *p* = 0.04, Cohen’s *F*^2^ = 0.06). The difference for the CD56^bright^CD16+ NK cells was in the same direction but became marginal after the BH correction was applied (PI: 0.79%, NA: 1.14%; uncorrected *p* = 0.03; BH corrected *p* = 0.06, Cohen’s *F*^2^ = 0.05).

The most common NK cells in circulation were the CD56^dim^CD16+ subset, accounting for between 90 and 92% of the total NK pool (Table 2). Until additional cell surface markers were considered, the percentiles for the CD56^dim^ subset (both CD16− and CD16+) did not differ significantly between the two rearing conditions (Figure 1C,D). Adolescents from both rearing conditions also had a substantial number of NK cells with minimal expression of the CD56 surface protein, but the relative abundance of these CD56−CD16+ NK cells did not differ between the two rearing conditions (mean percent of 9.4% and 10.2% for PI and NA, respectively, Table 2 and Figure 1E).

### 3.4. Effect of Early Rearing on Co-Expression of NKG2C and CD57

**Figure 2:**
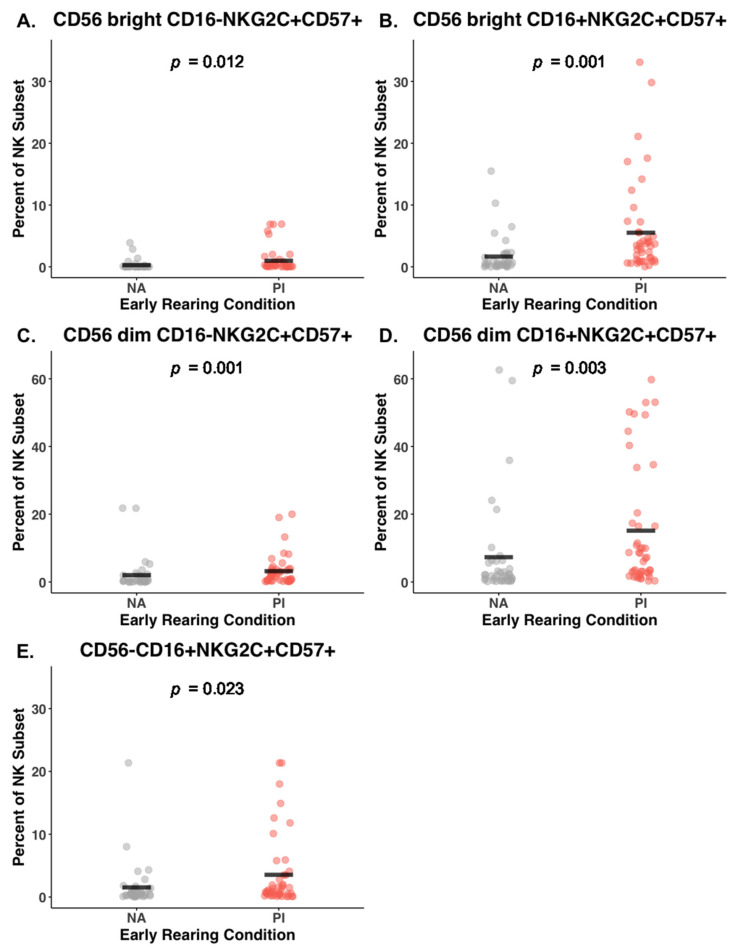
Co-expression of NKG2C and CD57 surface markers on the five primary NK subsets in non-adopted (NA) and post-institutionalized (PI) adolescents. Mean values are identified by horizontal black bars. Percentages were determined with respect to NK cells that were first gated and identified as being in the parent lineage. For each of the five subsets, NKG2C and CD57 were significantly more likely to be expressed on the NK cells of PI than NA adolescents (Panels **A**–**E**). Further analysis indicated that latent infection with CMV was common among the PI participants and was a contributing factor.

The gating strategy allowed us to differentiate the five primary NK subsets further based on co-expression of NKG2C and CD57 glycoproteins indicative of prior activation and stage of cell maturation. A history of institutionalization during infancy was associated with increased expression of NKG2C and CD57 on most of the CD56^bright^ and CD56^dim^ lineages (Table 2). Of particular note, both the CD56^dim^CD16− and CD56^dim^CD16+ NK lineages in the blood of PI participants also had significantly more cells co-expressing both of these additional surface markers (CD56^dim^CD16−NKG2C+CD57+ NK cells, 3.19% versus 2.01%; BH corrected *p* = 0.001, Cohen’s *F*^2^ = 0.21; and CD56^dim^CD16+NKG2C+CD57+ NK cells: 15.10% versus 7.27%; BH corrected *p* = 0.003, Cohen’s *F*^2^ = 0.20, respectively). This evidence for more mature cells with a history of activation in the PI adolescents was supported by another NK cell lineage: more of the NK cells with downregulated CD56 also co-expressed NKG2C and CD57 (CD56−CD16+NKG2C+CD57+ NK cells: 3.54% vs. 1.53% for PI and NA participants, respectively; BH corrected *p* = 0.02, Cohen’s *F*^2^ = 0.04) (Figure 2E).

### 3.5. Mediating Effect of CMV Seropositivity on CD56^dim^CD16+ NK Cells

**Figure 3:**
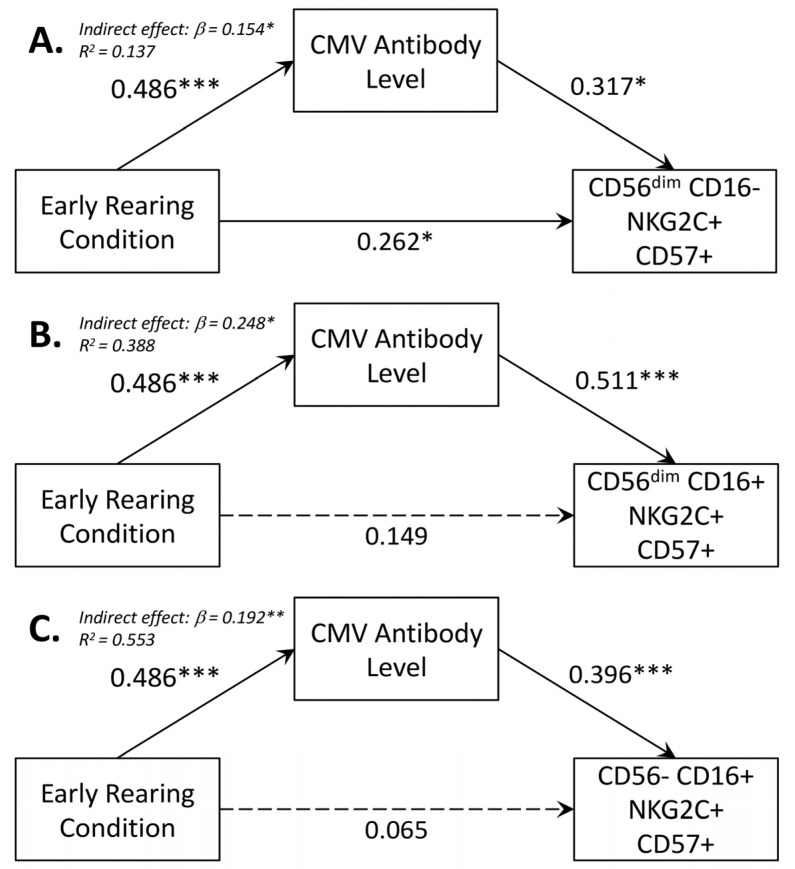
Path models examining the contribution of CMV infection to the association between early rearing condition and co-expression of NKG2C and CD57 on three different NK cell subsets. (**A**) Expression of NKG2C and CD57 on CD56^dim^CD16− NK cells. (**B**) Expression of NKG2C and CD57 on CD56^dim^CD16+ NK cells, the predominant NK cell subset in blood. (**C**) Expression of NKG2C and CD57 on NK cells with downregulated CD56 (CD56−-CD16+). * *p* < 0.05, ** *p* < 0.01, *** *p* < 0.001; More details on these path models, including β values and confidence intervals, are provided in Table 3.

As reported previously, PI adolescents were more likely to be CMV-seropositive (41 of 45) than the control participants in the NA rearing condition (5 of 39) [30]. They also had significantly higher levels of CMV-specific IgG (Table 1). To explore the potential influence of CMV status on the NK cell profiles, several path models were generated, focusing on the predominant NK cells in circulation, which were in the CD56^dim^ population; specifically, on the CD56^dim^ NK cells that co-expressed NKG2C and CD57. This modeling identified a potentiating influence of latent CMV infection, which contributed to the effect of early rearing on this NK cell subset (Figure 3). In the first path model, CMV antibody level statistically mediated the effect of early rearing on the CD56^dim^CD16−NKG2C+CD57+ NK cells (β_indirect_ = 0.154, SE = 0.079, bootstrapped 95% CI [0.028, 0.337]) (Figure 3A). In a second model, CMV antibody level fully mediated the effect of early rearing on the CD56^dim^CD16+NCG2C+CD57+ NK cells (β_indirect_ = 0.248, SE = 0.069, bootstrapped 95% CI [0.132, 0.398]) (Figure 3B). Finally, a third path model examined whether CMV seropositivity statistically mediated the influence of early rearing on the presence of NKG2C and CD57 on NK cells with downregulated CD56 expression (Figure 3C). The latter analysis of CD56−CD16+NCG2C+CD57+ NK cells also indicated that CMV status was a significant contributor (β_indirect_ = 0.192, SE = 0.072, bootstrapped 95% CI [0.072, 0.356]). Effect sizes and *p*-values for these three path models are provided in Table 3.

### 3.6. Effect of Early Rearing and CMV Status on Inflammatory Cytokines

**Figure 4:**
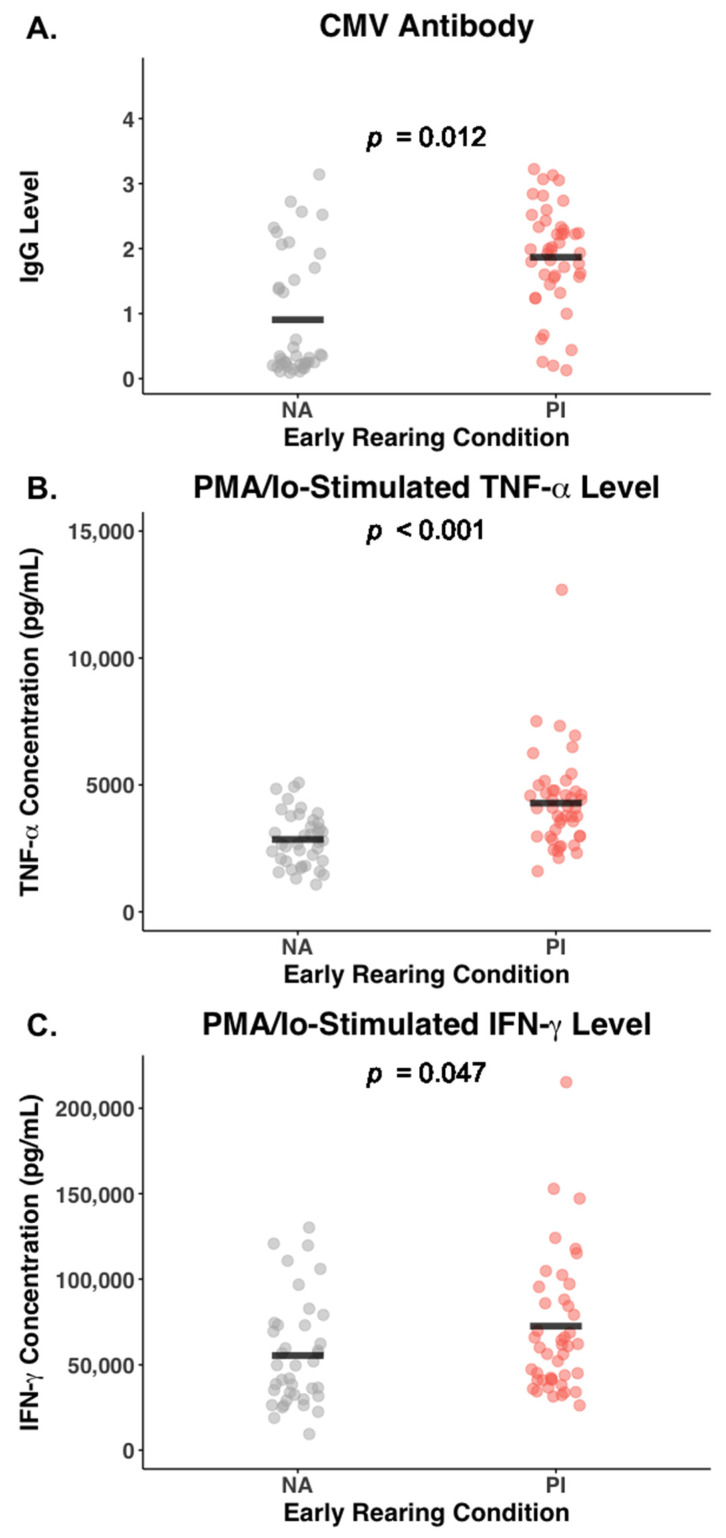
Three biomarkers considered as potential mediators of the effect of early rearing on the NK cells of non-adopted (NA) and post-institutionalized (PI) adolescents. (**A**) Blood levels of CMV-specific IgG indicating that more PI adolescents were CMV-seropositive with significantly higher antibody levels. (**B**) Significantly more TNF-α was released after stimulating the blood leukocytes from PI participants with a PMA/Io cocktail for 4–5 h. (**C**) Significantly more IFN-γ was also released into the cell supernatants after stimulating the PBMC of PI participants with the PMA/Io cocktail.

Adolescents who had been institutionalized during infancy had significantly higher blood levels of TNF-α than the NA adolescents (1.67 [0.65] vs. 1.32 [0.53] pg/mL; *F* (1,82) = 7.11, *p* = 0.009, η^2^ = 0.08). Their leukocytes also released markedly higher amounts of TNF-α after being cultured and stimulated with the PMA/Io cocktail (4295.3 [1876.6] vs. 2851.5 [1053.6] pg/mL; *F* (1,82) = 17.87, *p* < 0.001, η^2^ = 0.18). Basal blood levels of IFN-γ were similar in the two groups of adolescents (*F* (1,82) = 0.71, *p* = 0.40, η^2^ = 0.01), but the high PMA/Io-stimulated IFN-γ release was also indicative of more cellular reactivity in the PI adolescents (72,631.1 [44,700.3] vs. 55,430.8 [31,183.1] pg/mL, respectively; *F* (1,82) = 4.06, *p* = 0.05, η^2^ = 0.05). The significant effects of early institutional rearing on PMA/Io-induced secretion of TNF-α and IFN-γ, along with the significant difference in CMV-specific IgG levels, are shown in Figure 4.

Mediation models were generated to examine whether CMV status might account for the parallel effects of adverse rearing on both cytokine activity and NK cells. They did affirm that CMV-specific IgG level was statistically associated with the higher PMA/Io-stimulated TNF-α release in PI participants (β_indirect_ = 0.187, SE = 0.053, bootstrapped 95% CI [0.092, 0.298]). CMV-specific IgG level also statistically mediated the effect of early institutionalization on the higher PMA-stimulated IFN-γ release from the cultured leukocytes of PI participants (β_indirect_ = 0.173, SE = 0.061, bootstrapped 95% CI [0.069, 0.306]). CMV antibody level appeared to be associated with blood TNF-α levels in bivariate correlations (*r* = 0.499, *p* < 0.001), but the mediation models did not indicate that it accounted for the effect of early institutionalization on basal TNF-α levels (β_indirect_ = 0.072, SE = 0.052, bootstrapped 95% CI [−0.026, 0.182]). Similarly, mediation models did not discern an effect of CMV seropositivity on the basal IFN-γ levels in the blood (β_indirect_ = −0.040, SE = 0.038, bootstrapped 95% CI [−0.097, 0.060]).

**Figure A2:**
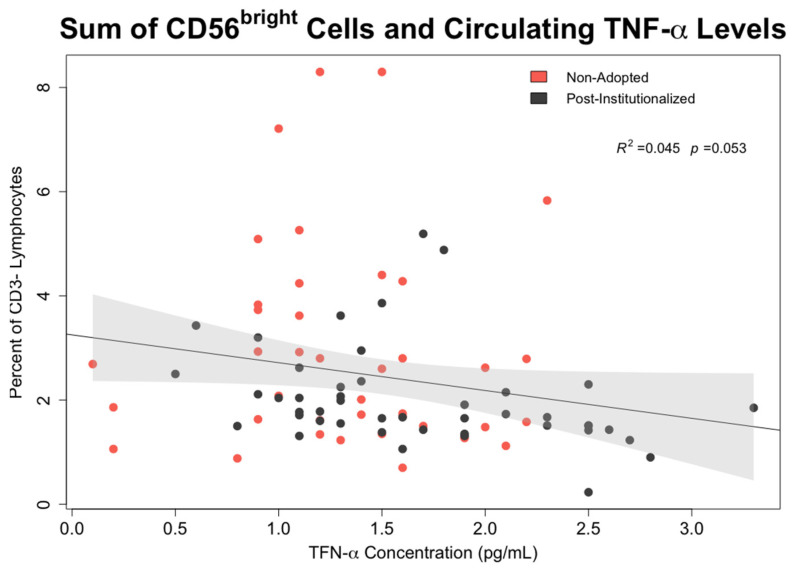
Association between blood TNF-α levels and the percent of CD56^bright^ NK cells in the blood of PI and NA adolescents. The sum includes both CD56^bright^CD16− and CD56^bright^CD16+ NK lineages. The relationship between these NK cells and PMA/Io-induced TNF-α level was also inverse and marginally significant. High levels of both TNF-α and IFN-γ stimulated by PMA/Io were negatively associated with the percent of CD56^bright^ NK cells in circulation (*r* = −0.22 and −0.23, *p* < 0.05, respectively).

**Figure A3:**
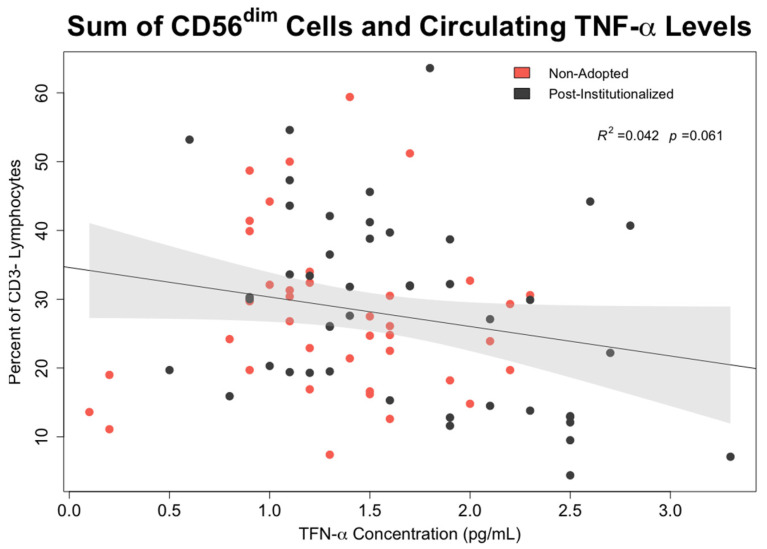
Association between blood TNF-α levels and the percent of CD56^dim^ NK cells in the blood of the PI and NA adolescents. The sum includes both CD56^dim^CD16− and CD56^dim^16+ NK lineages. The relationship between these NK cell populations and PMA/Io-induced TNF-α level was inverse and of a small magnitude (*r* = −0.15, *N.S.*). High IFN-γ levels stimulated by PMA/Io were negatively and marginally associated with the percentage of CD56^dim^ NK cells in circulation (*r* = − 0.14, *p* < 0.06).

Additional analyses examined whether basal levels of TNF-α and IFN-γ in the blood or the amount of cytokine elicited by PMA/Io stimulation were predictive of the relationship between CMV and the NK cell repertoire. However, the correlations between cytokine levels and types of NK cells in circulation were only modest. High TNF-α levels, both in the blood and after in vitro stimulation, were only marginally and inversely associated with the percentiles of CD56^bright^ and CD56^dim^ cells (r = −0.20 to −0.22) (Figure A2 and Figure A3 in Appendix A). The negative and low correlations did not indicate that NK cells were the primary cellular source of TNF-α. Conversely, they suggested that high levels of proinflammatory activity could have an inhibitory effect on the types of NK cells in the blood.

## 4. Discussion

Examining the leukocytes of youth adopted from orphanage-like institutions revealed long-term immune sequelae of early care during infancy and provided the unique opportunity to distinguish those effects from the consequences of adversity that occurred later throughout childhood or during adolescence. Typically, such a dramatic shift from a negative to more supportive environmental context does not occur in children born into families contending with difficult, low-resourced circumstances [69,70].

Immunophenotyping the NK cells of adopted adolescents identified several significant alterations that appeared to be associated with their early rearing experiences. Specifically, the CD56^bright^ NK cells were present at a lower level in the blood of the PI adolescents. In addition, most PI adolescents were CMV-seropositive, which was associated with NKG2C and CD57 surface markers being expressed on more NK cells, including the CD56^dim^ lineages that predominate in blood. Nevertheless, these quantitative differences in NK cells circulating in the blood stream were manifest within the context of an overall lymphocyte repertoire that appeared to be mostly normal and resilient.

The lower percent of CD56^bright^ NK cells likely reflects a shift in the distribution and trafficking patterns of NK cells, with more CD56^bright^ NK cells remaining resident in tissue [71,72,73,74]. This NK cell subset can also egress from the vasculature more readily than other NK cells and return back to tissue, including to lymph nodes and the liver. Alternatively, because the CD56^bright^ NK cells are thought to be less mature and can serve as precursors of the CD56^dim^ lineages, a reduction in CD56^bright^ NK cells may be indicative of a smaller reserve pool. An effect of early rearing on the proliferation kinetics of NK cells would be an important observation because it is estimated that about 4% of the body’s entire NK population turns over each day, and the life span of a mature NK cell can be as short as 2–3 weeks [75]. The potential clinical significance of an imbalance of CD56^bright^ and CD56^dim^ NK populations can be seen more clearly in pediatric cancer patients who have had their lymphoid tissue and cells ablated with immunosuppressive treatments. Following the administration of hematopoietic stem cell transplants, the CD56^bright^ NK cells recover several weeks earlier than the CD56^dim^ lineages, which supports the view that they can emerge earlier in the serial sequence of NK cell maturation [76].

It should be reiterated that the most common NK cells in the blood, the CD56^dim^CD16+ subset, appeared initially to be within the normal range and did not seem to be impacted by the adverse early rearing of the PI adolescents. It was only when the NK cells were differentiated further by examining the co-expression of NKG2C and CD57 surface markers that an important influence of early rearing became apparent for both the CD56^bright^ and CD56^dim^ NK lineages. This effect on NK cells was accentuated by the high prevalence of CMV infection among the PI adolescents. CMV is known to result in an expansion of NK cells expressing both NKG2C and CD57 in both healthy adults and patients [77,78,79]. The presence of both NKG2C and CD57 is a cell surface phenotype associated with a history of activation and replication and denotes a mature NK cell that may be in a stage of terminal differentiation. While mature NK cells can still respond in a dynamic fashion to immune challenges and readily engage in cytolytic responses, there are homeostatic set points and regulatory feedback that govern their proliferation rate in a healthy individual [80,81]. Specifically, CMV seropositivity was associated with an increased percent of terminally differentiated NK cells in the blood of PI adolescents. It is likely that early infections with pathogens during infancy, including haemophilus influenzae and herpes viruses, magnify the impact of psychosocial deprivation on the developing immune system [82,83]. Exposure to CMV is known to be common in infants and children raised in larger groups and is thus important to consider when evaluating immunity in PI individuals [37]. However, because it is difficult to find PI youth who have not been infected with CMV, it is challenging to disentangle the immune effects of a deprived early rearing from the cumulative toll of herpes viruses on the immune system.

**Figure A4:**
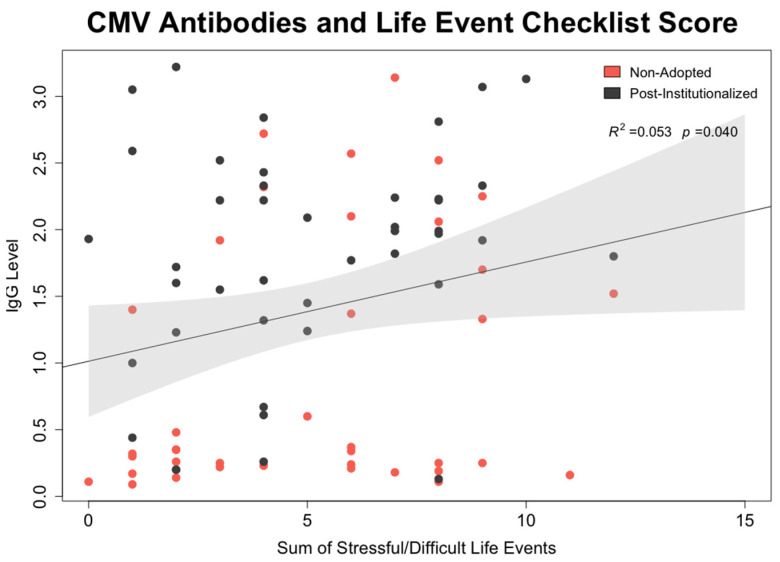
Association between CMV antibody level and the Life Events Checklist (LEC) score for the PI and NA adolescents. There was a significant relationship between experience of traumatic and stressful life events and CMV antibody titer (*r* = 0.23, *p* = 0.04). Higher CMV antibody was associated with the occurrence of more difficult life events among adolescent participants in both groups.

**Figure A5:**
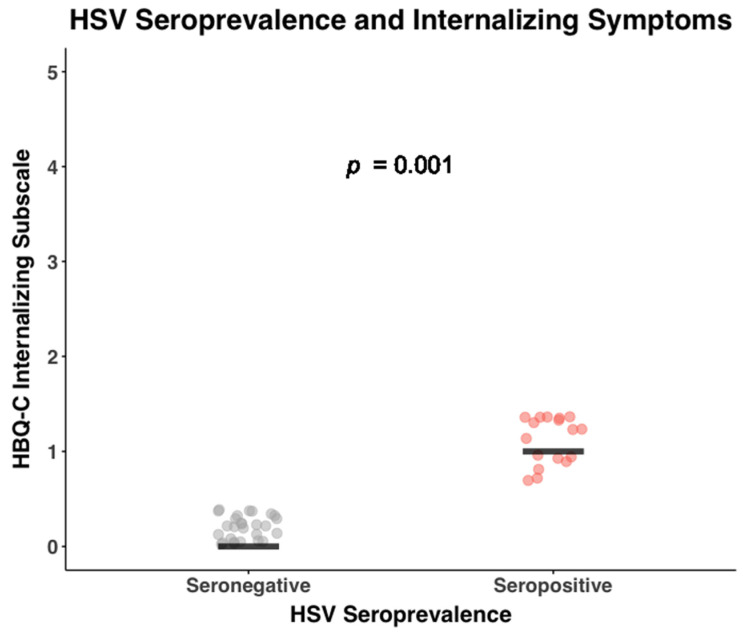
Association between HSV seroprevalence and the internalizing symptom subscale from the Health and Behavior Questionnaire (HBQ-C). Adolescents who had high antibody titers above the threshold to be considered HSV-seropositive self-reported more depressive and anxious symptoms (*t* (85) = −3.25, *p* = 0.002).

Research on the immune effects of ELA in adolescents has sometimes yielded inconsistent findings because of the many different psychosocial and physiological factors that can influence immunity. For example, while most studies have found that there is a reduction in lymphocyte proliferative responses and cytolytic activity after exposure to adversity, some researchers have reported the opposite effect in adolescents [84,85,86,87,88]. Some inconsistencies may be due to not accounting for variation in the number and types of lymphocytes present in the blood, which affects how cells will respond when cultured with stimulants and how effectively they lyse target tissue [89]. For example, we found that about 10% of the NK cells in the adolescent participants had downregulated CD56 expression. CD56−CD16+ NK cells are less responsive to stimulation with cytokines like IFN-γ and could appear to have lower effector function. Such a high percent of NK cells with downregulated CD56 expression was an unexpected observation because elevations of this lineage are more typically seen in individuals with chronic infections, including in children with malaria and adults with hepatitis, AIDS, and tuberculosis [90]. Another likely explanation for the inconsistent findings on ELA and immunity in adolescents is that it matters whether the adverse conditions are still ongoing or have resolved. Studies that found evidence for increased proliferative and cytolytic activity have typically evaluated adolescents who were still living in impoverished and stressful conditions. These participants often have high levels of depression and anxiety. In contrast, the atypical rearing situation in our study had been resolved for more than a decade prior to the current assessment. In addition, at the time of blood collection, we did not find group differences in either the occurrence of other traumatic events or the current experiencing of negative emotions. The only linkage between psychosocial stress and immunity was seen with respect to herpes viruses, not with the NK cell repertoire. Higher levels of CMV antibody were positively correlated with the number of traumatic and stressful life events the participants had experienced (Figure A4), and internalizing symptoms were higher among the HSV-seropositive participants from both rearing conditions (Figure A5). The effect of stressful life events and negative emotions on antibody titers and the reactivation of herpes viruses concurs with the findings of many studies [91,92]. In contrast, the differences in the NK cells appeared to be due more specifically to the early rearing experiences during infancy. They affirm the importance of supportive and nurturing parental care for promoting neurobehavioral development, emotional well-being, and immune health.

Further research is needed to understand which physiological systems retain the resiliency and capacity to recover when life circumstances improve. It may not be possible to completely undo some of the harmful consequences of ELA, including the deleterious effects on brain structure and function that have been identified [93,94,95]. It is not known if the parallel effects of ELA on other physiological systems, including the immune system, contribute to the developmental effects on the brain, nor whether hormonal or immunomodulatory treatments could be helpful as therapeutic modalities [96]. The NK cells characterized in our study would not directly affect the brain. NK cells do not readily cross the blood–brain barrier (BBB), and the number of NK cells in the central nervous system is low in a healthy individual [97,98]. There are normally only a few hundred NK cells present in a mL of cerebrospinal fluid, although that number can increase if there is a breakdown in the BBB or following traumatic injury, central nervous system infections, or a cancerous growth in the brain [99]. It may also be important that the NK cell subsets in CSF are skewed toward the CD56^bright^ NK lineages, unlike the predominance of CD56^dim^ NK cells in the blood. Activated CD56^bright^ NK cells are more prone to synthesize and release cytokines and chemokines, and an elevated release of these soluble proteins in the intrathecal compartment could potentially influence neural activity.

### Study Limitations

Notwithstanding the potential implications and novelty of our findings, several limitations should be acknowledged. The PI adolescents were recruited in a standardized manner but were adopted from many different countries, and the age at adoption varied. Preliminary analyses indicated that adoption age did not significantly affect the NK cell populations, and thus it was not included as a control variable in the statistical models. There was also variation in age at the time of blood collection. Even though the numbers and types of NK cells in blood are known to be adult-like and stable by the time of puberty [100,101], participant age was included as a covariate because many other factors do change across adolescence. We did not see a significant effect of participant age at blood draw on any of the NK cell subsets. Due to the limitations of sample size, we did not probe for interactions between early rearing and sex of the participant. Instead, sex of the participant was included as covariate in the statistical modeling, and we ensured that gender representation was similar in each rearing condition. The possibility of a differential vulnerability of males and females to ELA should be addressed in future studies with larger sample sizes (the mean values of the NK cell indices and other biomarkers for females and males are provided in Table A1). Similarly, due to the limited sample size and limited racial diversity among the NA participants, the potential influence of race and ethnicity was not considered. It would have required dichotomizing the participants into just two groups, White vs. non-White. In addition, the racial diversity was not evenly distributed across the two rearing conditions, raising a concern about multicollinearity in the modeling. Future research should consider the possibility of racial and ethnic group variation in the extent of immune vulnerability in different populations. Finally, our NK cell measures were based on immunophenotyping and did not include specific tests of function. We attempted to employ two prominent cytokines secreted by NK cells, TNF-α and IFN-γ, as proxy indicators of NK activation and effector function after PMA/Io stimulation. However, the inverse relationship between cytokine levels and the NK cell indices did not support the interpretation that NK cells were the primary source of cytokines present in the blood or cell supernatants.

## 5. Conclusions

In summary, our findings concur with many studies that have found adverse and impoverished early rearing conditions result in long-lasting physiological effects [102,103]. The persistent alterations are also in keeping with neurobehavioral evaluations that continue to raise concerns about the communal rearing of infants in institutions and convey the benefits of foster parenting and adoption [104,105]. The adolescents in our study likely benefited from being adopted into supportive families for over a decade prior to the current assessment. The most salient effects on the NK cell repertoire appeared to be attributable to early CMV infections, which led to an expansion of NK cells co-expressing NKG2C and CD57. Being CMV-seropositive was also associated with higher levels of TNF-α in the blood, as well as more TNF-α and IFN-γ released after leukocyte stimulation. This effect of CMV seropositivity on immunity is in keeping with previous research showing that it can also affect the protective responses elicited by vaccination [106]. Cytokine levels, along with many other stress-responsive mediators released by the neuroendocrine axis and sympathetic nervous system, can affect the trafficking and functioning of NK cells [107,108,109]. Increased inflammatory activity appeared to have an inhibitory effect on the NK cell populations in circulation. The sensitivity of the NK cell repertoire to early life events may also reflect that set points for the proportions of CD56^bright^ and CD56^dim^ NK cells in the blood become established during infancy and already transition to the mature adult-like profile in young children [110,111]. Research on young monkeys and piglets [112,113] has shown further that other experiential factors during infancy, including consumption of breast milk or formula, can affect the numbers and types of NK and T cells in a developing infant.

## Figures and Tables

**Table 1:** Descriptive statistics for the post-institutionalized (PI) and non-adopted (NA) participants. Means (SD) are shown for primary variables used in the analyses.

	PI (*n* = 45)	NA (*n* = 39)	*p*-Value ^2^
Mean age (yr)	16.6 (2.2)	16.0 (1.7)	0.15
Female, *n* (%) ^1^	25 (55.6%)	22 (56.4%)	0.94
LEC score	5.07 (2.95)	5.08 (3.17)	**0.99**
HBQ-C score	2.32 (0.68)	2.17 (0.68)	**0.30**
Height (cm)	163.6 (9.2)	170.6 (10.1)	**0.001**
Weight (kg)	60.5 (13.3)	64.8 (16.7)	0.20
WBC (10^3^ per µL)	6.16 (1.66)	5.87 (1.59)	0.39
Lymphocyte (%)	34.80 (9.49)	31.91 (7.07)	0.07
Neutrophil (%)	54.98 (10.84)	57.48 (8.23)	0.14
CD3− (% of Lymph)	36.48 (12.29)	36.03 (13.40)	0.79
NK (% of Lymph)	15.64 (10.98)	15.48 (10.72)	0.92
CD56^bright^ (% of CD3−)	2.03 (0.96)	2.93 (1.96)	**0.008**
CD56^dim^ (% of CD3−)	28.56 (13.92)	27.65 (11.85)	0.75
CMV titer	1.87 (0.79)	0.91 (0.94)	**0.001**
HSV titer	0.99 (1.34)	0.53 (1.10)	0.06
Blood TNF-α (pg/mL)	1.75 (0.62)	1.33 (0.56)	**0.009**
Blood IFN-γ (pg/mL)	3.95 (3.73)	6.46 (3.15)	0.66
PMA/Io TNF-α (pg/mL)	4269.3 (1895.1)	2847.5 (1067.5)	**0.001**
PMA/Io IFN-γ (pg/mL)	72,631.1 (44,700.3)	55,430.8 (31,183.1)	**0.047**
CRP (mg/L)	2.24 (4.07)	1.75 (3.06)	0.64

^1^ Sex distribution analyzed with the chi-squared test. ^2^ Significant *p* values are highlighted with bold font. *Note*: Participant race and ethnicity were not considered in our models due to the relatively small sample size and the racial homogeneity of the NA group (89.7% White). Only a comparison of White vs. non-White would have been possible, and there was a statistical concern about multicollinearity given the overlap with the rearing variable.

**Table 2:** Differences in NK cell repertoires of post-institutionalized (PI) and non-adopted (NA) adolescents (*N* = 84).

Cell Type	PI (*n* = 45) %	NA (*n* = 39) %	Rearing Coeff. ^1^ (SE)	95% CI	*p*	Corr. *p* ^2^	Cohen’s ^3^ (*F*^2^)
**Sum NK (% CD3−)**							
CD56+proportion	92.20 (0.41)	90.00 (0.58)	0.24 (0.10)	(0.05, 0.43)	**0.01**	**0.03**	0.07
Sum CD56^bright^	2.03 (0.96)	2.93 (1.96)	−0.28 (0.10)	(−0.46, −0.09)	**0.004**	**0.02**	0.08
Sum CD56^dim^	28.56 (13.92)	27.65 (11.85)	−0.00 (0.11)	(−0.21, 0.21)	0.97	0.99	0.00
**CD56** **^bright^CD16−**	1.24 (0.51)	1.73 (1.09)	−0.23 (0.11)	(−0.43, −0.03)	**0.02**	**0.04**	0.06
NKG2C−CD57−	92.78 (7.82)	95.01 (3.85)	−0.19 (0.90)	(−0.35, −0.03)	**0.02**	**0.04**	0.04
NKG2C−CD57+	2.12 (4.99)	0.91 (2.43)	0.20 (0.11)	(−0.02, 0.41)	0.07	0.10	0.04
NKG2C+CD57−	4.01 (2.74)	3.82 (2.85)	0.18 (0.10)	(−0.00, 0.36)	0.05	0.08	0.03
NKG2C+CD57+	0.98 (2.00)	0.27 (0.79)	0.30 (0.10)	(0.10, 0.50)	**0.003**	**0.01**	0.11
**CD56** **^bright^CD16+**	0.79 (0.58)	1.14 (0.98)	−0.22 (0.10)	(−0.42, −0.02)	**0.03**	0.06	0.05
NKG2C−CD57−	77.97 (12.46)	84.96 (10.06)	−0.29 (0.10)	(−0.47, −0.10)	**0.002**	**0.01**	0.10
NKG2C−CD57+	7.62 (7.01)	4.04 (4.33)	0.24 (0.09)	(0.06, 0.41)	**0.008**	**0.02**	0.06
NKG2C+CD57−	9.46 (5.76)	9.31 (7.58)	0.22 (0.90)	(0.09, 0.37)	**0.006**	**0.02**	0.05
NKG2C+CD57+	5.52 (7.52)	1.66 (3.09)	0.38 (0.10)	(0.19, 0.56)	**0.000**	**0.001**	0.17
**CD56** **^dim^CD16−**	0.73 (0.28)	0.73 (0.40)	0.05 (0.11)	(−0.16, 0.26)	0.64	0.99	0.00
NKG2C−CD57−	78.43 (13.28)	84.87 (10.48)	−0.23 (0.11)	(−0.44, −0.02)	**0.03**	0.06	0.06
NKG2C−CD57+	10.80 (10.12)	8.29 (7.72)	0.24 (0.11)	(0.04, 0.44)	**0.02**	**0.04**	0.06
NKG2C+CD57−	5.30 (4.17)	4.21 (3.57)	0.26 (0.08)	(0.11, 0.40)	**0.000**	**0.001**	0.08
NKG2C+CD57+	3.19 (4.44)	2.01 (4.85)	0.42 (0.09)	(0.25, 0.59)	**0.000**	**0.001**	0.21
**CD56** **^dim^CD16+**	27.82 (13.82)	26.92 (11.97)	0.00 (0.11)	(−0.21, 0.21)	0.99	0.77	0.00
NKG2C−CD57−	33.61 (14.06)	43.64 (17.26)	−0.30 (0.10)	(−0.49, −0.11)	**0.002**	**0.01**	0.10
NKG2C−CD57+	48.08 (16.04)	47.14 (17.29)	0.03 (0.12)	(−0.20, 0.25)	0.83	0.92	0.00
NKG2C+CD57−	3.06 (2.80)	1.95 (1.96)	0.25 (0.10)	(0.05, 0.45)	**0.02**	**0.04**	0.07
NKG2C+CD57+	15.10 (18.20)	7.27 (14.62)	0.40 (0.10)	(0.21, 0.59)	**0.000**	**0.003**	0.20
**CD56−CD16+**	9.40 (7.17)	10.19 (9.49)	−0.05 (0.11)	(−0.26, 0.16)	0.63	0.79	0.00
NKG2C−CD57−	74.10 (13.12)	77.14 (13.06)	−0.12 (0.11)	(−0.33, 0.09)	0.26	0.36	0.02
NKG2C−CD57+	11.36 (10.46)	12.101 (1.011)	0.08 (0.11)	(−0.20, 0.23)	0.88	0.94	0.00
NKG2C+CD57−	10.52 (10.44)	9.34 (8.38)	−0.04 (0.11)	(−0.25, 0.18)	0.75	0.87	0.00
NKG2C+CD57+	3.54 (5.72)	1.53 (3.59)	0.26 (0.10)	(0.07, 0.45)	**0.008**	**0.02**	0.04

*Note.* Mean (SD) values are shown for five major NK subsets and two additional surface proteins. Bolded *p* values indicate *p* < 0.05. ^1^ Rearing effect tested with log 10 transformed values, adjusted for sex, age, and time of blood collection. PI = 1 and NA = 0. All estimates were calculated with the robust maximum likelihood estimator. ^2^ Benjamini–Hochberg corrected *p*-value. ^3^ Effect size: significance of including rearing condition as a predictor of differences in NK cells in models.

**Table 3:** Indirect effects of latent CMV infection on NK cell repertoire in post-institutionalized (PI) and non-adopted (NA) adolescents (*N* = 84).

	CMV-IgG Level on Early Rearing		NK Cell Subsets on CMV-Specific IgG		NK Cell Subset on Early Rearing ^1^					
NK Cell	β (SE) ^2^	95% CI ^3^	β (SE)	95% CI	Total Effect	95% CI	Direct Effect	95% CI ^3^	Indirect Effect	95% CI
CD56^dim^ CD16− NKG2C+ CD57+	**0.486 (0.092)**	**(0.301, 0.660)**	**0.317 (0.131)**	**(0.067, 0.580)**	**0.416 (0.091)**	**(0.223, 0.580)**	**0.262 (0.031)**	**(0.004, 0.477)**	**0.154 (0.079)**	**(0.028, 0.337)**
CD56^dim^ CD16+ NKG2C+ CD57+	**0.486 (0.092)**	**(0.301, 0.660)**	**0.511 (0.086)**	**(0.347, 0.683)**	**0.398 (0.099)**	**(0.196, 0.581)**	0.149 (0.112)	(−0.077, 0.361)	**0.248 (0.069)**	**(0.132, 0.398)**
CD56−CD16+ NKG2C+ CD57+	**0.486 (0.092)**	**(0.301, 0.660)**	**0.396 (0.114)**	**(0.172, 0.621)**	**0.258 (0.101)**	**(0.055 0.450)**	0.065 (0.127)	(−0.193, 0.313)	**0.192 (0.072)**	**(0.072, 0.356)**

*Note.* Bolded numbers indicate *p* < 0.05. ^1^ Early rearing conditions are PI (0) or NA (1). ^2^ Coefficient (SE). ^3^ All estimates and 95% confidence interval calculations were based on 10,000 bootstrapped samples.

## Data Availability

Access to original data can be requested by appropriately authorized professionals but will not include information that could be used to identify individuals.

## Appendix A

**Table A1:** Immune indices and other biomarkers, including those suggestive of similarities or differences between females and males or evincing a trend for a sex-related differential in ELA effects.

*Percent of NK Cell Subsets*				
	Female	Male	Sex	Sex X Rearing Interaction
	*M* (SD)	*M* (SD)	*p*	*p*
Sum CD56 Proportion	91.50 (4.84)	90.85 (5.41)	0.37	0.09
Sum CD56^bright^	2.35 (1.48)	2.56 (1.68)	0.36	0.21
Sum CD56^dim^	27.70 (13.12)	28.69 (12.84)	0.83	0.48
**CD56** **^bright^** **CD16−**	1.48 (0.87)	1.45 (0.86)	0.99	0.48
NKG2C−CD57−	95.02 (5.87)	92.29 (4.61)	0.06	0.80
NKG2C−CD57+	1.18 (3.56)	2.05 (4.57)	0.31	0.67
NKG2C+CD57−	3.32 (2.39)	4.68 (3.07)	**0.04**	0.61
NKG2C+CD57+	0.47 (1.36)	0.89 (1.84)	0.26	0.32
**CD56** **^bright^** **CD16+**	0.85 (0.67)	1.07 (0.94)	0.14	0.15
NKG2C−CD57−	83.97 (11.28)	77.73 (12.36)	**0.02**	0.37
NKG2C−CD57+	5.31 (5.88)	6.79 (6.47)	0.27	0.64
NKG2C+CD57−	7.83 (5.62)	11.37 (7.32)	**0.02**	0.95
NKG2C+CD57+	2.86 (4.53)	4.83 (7.71)	0.20	0.06
**CD56^dim^** **CD16−**	0.76 (0.34)	0.69 (0.34)	0.44	0.96
NKG2C−CD57−	83.15 (11.96)	79.22 (12.80)	0.15	0.17
NKG2C−CD57+	9.73 (9.60)	12.16 (10.71)	0.23	0.99
NKG2C+CD57−	4.18 (3.59)	5.57 (4.22)	0.16	0.11
NKG2C+CD57+	2.43 (4.79)	2.92 (4.49)	0.70	0.11
**CD56^dim^** **CD16+**	26.94 (13.10)	28.00 (12.85)	0.81	0.48
NKG2C−CD57−	39.95 (16.77)	36.13 (15.71)	0.35	0.27
NKG2C−CD57+	47.91 (17.91)	47.31 (14.85)	0.93	0.41
NKG2C+CD57−	2.16 (2.25)	3.02 (2.74)	0.13	0.70
NKG2C+CD57+	9.82 (16.37)	13.54 (17.77)	0.43	0.07
**CD56−** **CD16+**	9.24 (8.74)	10.44 (7.72)	0.50	0.67
NKG2C−CD57−	79.06 (12.10)	71.01 (13.08)	**0.007**	0.63
NKG2C−D57+	11.32 (9.76)	12.19 (11.83)	0.75	0.46
NKG2C+CD57−	7.28 (6.46)	13.39 (11.55)	**0.004**	0.24
NKG2C+CD57+	2.30 (5.09)	2.99 (4.76)	0.63	0.53
	*M* (SD)	*M* (SD)	*p*	*p*
WBC (10^3^ per µL)	6.16 (1.49)	5.65 (1.73)	0.06	0.40
Lymphocyte (%)	31.34 (8.27)	36.14 (8.19)	**0.01**	0.16
Neutrophil (%)	59.64 (8.65)	51.70 (9.34)	**0.002**	0.15
NK (% of CD3−)	14.63 (12.10)	16.75 (8.89)	0.40	0.73
CMV titer	1.41 (1.01)	1.43 (0.97)	0.97	0.97
HSV titer	0.76 (1.25)	0.80 (1.27)	0.98	0.11
Blood TNF-α (pg/mL)	1.33 (0.56)	1.75 (0.62)	**0.005**	0.37
Blood IFN-γ (pg/mL)	5.64 (17.67)	4.45 (5.52)	0.47	0.41
PMA/Io TNF-α (ng/mL)	3.06 (1.06)	4.31 (2.11)	**0.001**	0.09
PMA/Io IFN-γ (ng/mL)	52.18 (23.00)	80.48 (50.04)	**0.02**	0.05
CRP (mg/L)	1.72 (2.91)	2.40 (4.42)	0.37	0.39

*Note.* Significant *p*-values are bolded. Age and time at blood draw were included as covariates in all analyses.
